# Clinical efficacy and safety of high-intensity focused ultrasound (HIFU) ablation in treatment of cesarean scar pregnancy (CSP) I and II

**DOI:** 10.1186/s12884-022-04848-z

**Published:** 2022-07-30

**Authors:** Yanglu Liu, Qiaozhi Yin, Fan Xu, Shuang Luo

**Affiliations:** 1 School of Medical and Life Sciences,Chengdu University of Traditional Chinese Medicine, 37 Twelfth Bridge Road, Chengdu, 610072 Sichuan China; 2 Chengdu Hospital for Reproduction, Women and Children, Chengdu University of Chinese Medicine, Sichuan Chengdu, China; 3 Department of Obstetrics and Gynecology, The Affiliated Nanchong Central Hospital of North Sichuan Medical College, Nanchong, Sichuan China; 4 Department of Obstetrics and Gynecology, Suining Central Hospital, Suinig, Sichuan People’s Republic of China

**Keywords:** High intensity focused ultrasound, Cesarean scar pregnancy, Ultrasound-guided dilation and curettage

## Abstract

**Objective:**

To investigate the safety and feasibility of high intensity focused ultrasound (HIFU) ablation followed by ultrasound-guided dilation and curettage (USg-D&C) for two types patients with cesarean scar pregnancy (CSP-I and CSP-II).

**Materials and methods:**

This study was a retrospective analysis of 101 CSP-I patients and 52 CSP-II patients who received HIFU ablation followed by USg-D&C from Jun 2014 to Oct 2020. The diameter of gestational sac/mass, thickness of the intervening myometrium, intraoperative blood loss, operation time, length of hospital stays, adverse effects and β-HCG level in the two groups were compared.

**Results:**

All patients successfully received HIFU ablation under conscious sedation. The median total treatment time of HIFU ablation and median USg-D&C time in the CSP-I group were statistically longer than those in the CSP-II group (*P <* 0.05). The average intraoperative median blood loss was 39 ml in the CSP-I group and 65 ml in the CSP-II group (*P <* 0.05). The duration of hospitalization was 7.07 ± 1.83 days in the CSP-I group and 7.18 ± 1.72 days in the CSP-II group (*P >* 0.05). The average time needed for β-HCG return to normal levels was 26.08 ± 5.02. and 28.15 ± 4.99 days for CSP-I and CSP-II, respectively (*P* > 0.05). The percentage of adverse effects and complications was not significantly different between the two groups (*P >* 0.05).

**Conclusions:**

HIFU ablation followed by USg-D&C was safe and effective in treating the CSP-I patients and CSP-II patients, which may be a potential noninvasive therapeutic option for patients with CSP.

## Introduction

Cesarean scar pregnancy (CSP) is a rare but potentially a devastating ectopic pregnancy, in which the gestational sac is implanted in a previous cesarean section scar and may cause life-threatening complications [1, 2]. Based on the location of gestational sac, there are two types of CSP: 1). In type-I (CSP-I),the amniotic sac is on the scar with progression of the pregnancy implanted in the isthmus uteri and in the uterine cavity; and 2). in Type II (CSP-II) the amniotic sac deeply implanted into previous cesarean scar defect with infiltrating growth into the uterine myometrium and bulging from the uterine serosal surface [3]. The management of CSP by dilation and curettage (D&C) had a high risk of severe hemorrhage, so it is not a first-line therapeutic option for CSP [4]. Currently, some researchers investigated whether HIFU is safe in management of patients with CSP [5–8]. As a noninvasive therapeutic technique, HIFU has been widely used to treat benign lesion of uterus [9, 10]. However, to the best of our knowledge, the safety and efficacy of HIFU ablation for different types of CSP was not reported. Therefore, this study attempted to 1) evaluate the efficacy of two types of CSP treated by HIFU ablation followed by ultrasound-guided dilation and curettage (USg-D&C); 2) to explore the safety HIFU ablation used to treat patients either of the two types of CSP.

## Materials and methods

### Patients

Suining central hospital (SCH) is located in Southwest China and the HIFU Center was founded in 2010. Its main indications include uterine fibroids, adenomyosis, CSP., nearly 8000 patients have been treated in HIFU center. The team has completed more than 200 cases with CSP since 2014. This study was a retrospective analysis of 153 patients with CSP from the Obstetrics and Gynecology Department of SCH between June 2014 and October 2020 who treated by HIFU ablation combined with USg-D&C.This study was approved by the Ethics Committee of Suining Hospital. Because of the retrospective study design, informed consent could not be obtained from each patients. Instead of obtaining informed consent from each patient, we posted a notice about the study design and contact information at a public location in the hospital. The following points are the diagnostic criteria for CSP:(1) amenorrhea or vaginal bleeding, (2) hematuria with positive β-HCG, and (3) ultrasound findings confirming the diagnosis and magnetic resonance imagine (MRI)-based determinations of CSP type. The inclusion criteria were as follows [5–7, 11]: (1) patients had a history of cesarean section delivery; (2) patients had a history of amenorrhea and positive urine pregnancy test; (3) ultrasound and MRI-based confirmation of the CSP diagnosis based on the diagnostic criteria recommended by Godin.(4) gestational age less than 9 weeks, (5) complete clinical date and (6) the patients were treated with HIFU. The exclusion criteria were as follows: (1) patients with unstable vital signs; (2) had other treatments related to CSP. (3) unclear CSP diagnosis or (4) the incomplete clinical or follow up data.

### Pre-HIFU ablation preparation

All patients were required to undergo specific bowel preparation before HIFU ablation, which included ingesting liquid food and fasting for 12 hours pre-treatment and a mandatory enema in the morning of treatment day. The hair on the abdominal wall was shaved from the umbilicus to the upper margin of the pubic symphysis, and the area was degreased and degassed with 75% ethanol, and a urinary catheter was inserted to control the bladder volume 30 min before HIFU treatment.

### HIFU ablation

**Fig. 1:**
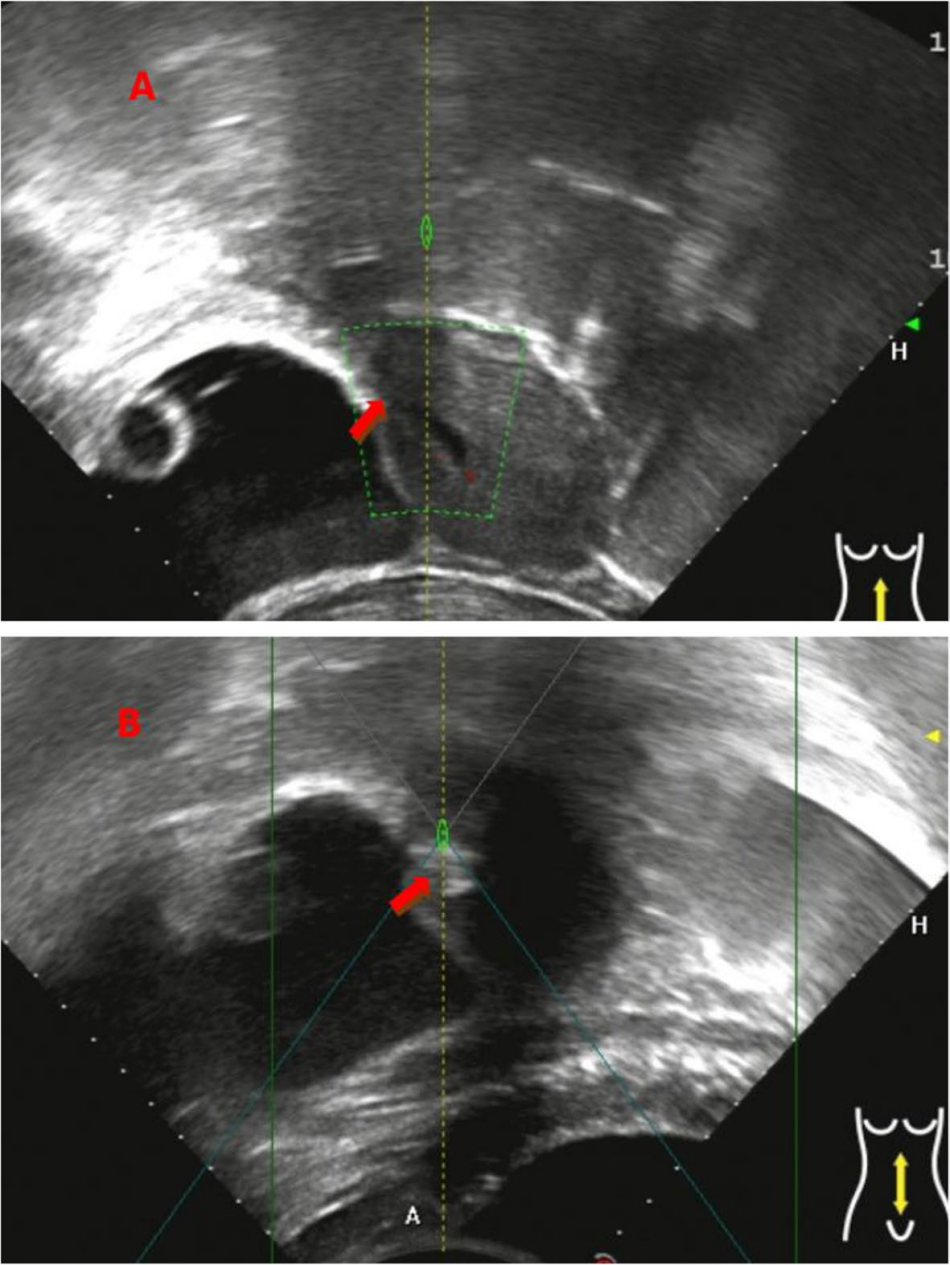
Ultrasound images obtained from a patient with CSP. **A** Pre-procedure contrast-enhanced ultrasound image shows the sac/mass (arrow). **B** Ultrasound image obtained immediately after HIFU ablation shows gray scale change at the target area (arrow)

HIFU ablation was performed under conscious sedation using a JC200 tumor therapeutic system (Chongqing HIFU Medical Tech Co, Ltd., Chongqing, China). A transducer, with a 20 cm diameter and a focal length of 15 cm, produced the therapeutic energy required for treatment. An ultrasound device (MyLab 70, Esaote, Genova, Italy) was used to provide real-time imaging for monitoring the response of HIFU treatment for CSP. Every patient was carefully positioned prone on the HIFU ablation bed with the anterior abdominal wall in contact with the degassed water balloon. The treatment plan was made by dividing the gestational sac into different slices with the thickness of 3 mm. The sonication power was 350-400 w, and the treatment was terminated when the gray scale change at the target tissue was observed or the signal of the blood flow of pregnancy tissue disappeared on ultrasound (Fig. 1). The blood pressure, pulse, respiration rate and peripheral oxygenation of patients were monitored continuously.

### USg-D&C

USg-D&C was performed under general anesthesia 3-6 days after HIFU ablation [5–7]. The patients was placed in lithotomy position. The ultrasound was used to locate the site of the pregnancy tissue in the myometrial defect. During the procedure, a 6- or 7-mm suction cannula was gently inserted into the uterine cavity and the vacuum pressure was set at 400 mmHg. The operator moved the cannula back and forth, rolling the cannula gently to detach the pregnancy tissues from the previous cesarean scar.

### Follow-up observation

The β-HCG level was monitored weekly until it returned to normal after discharge. The patients were requested to have ultrasound examination 1 month after treatment. The time of vaginal bleeding, abdominal pain, the first return of the menstrual cycle and successful pregnancy of patients with pregnancy were recorded.

### Statistical analysis

Statistical analysis was performed with SPSS 17.0 software. Continuous variables were summarized as the mean ± standard deviation if normally distributed, and discrete variables were described as median. Comparisons between the two groups were analyzed by the t test for continuous variables and the chi-square test for categorical data. A *P* value of less than 0.05 was considered to indicate a statistically significant difference.

## Results

### Baseline characteristics

**Fig. 2:**
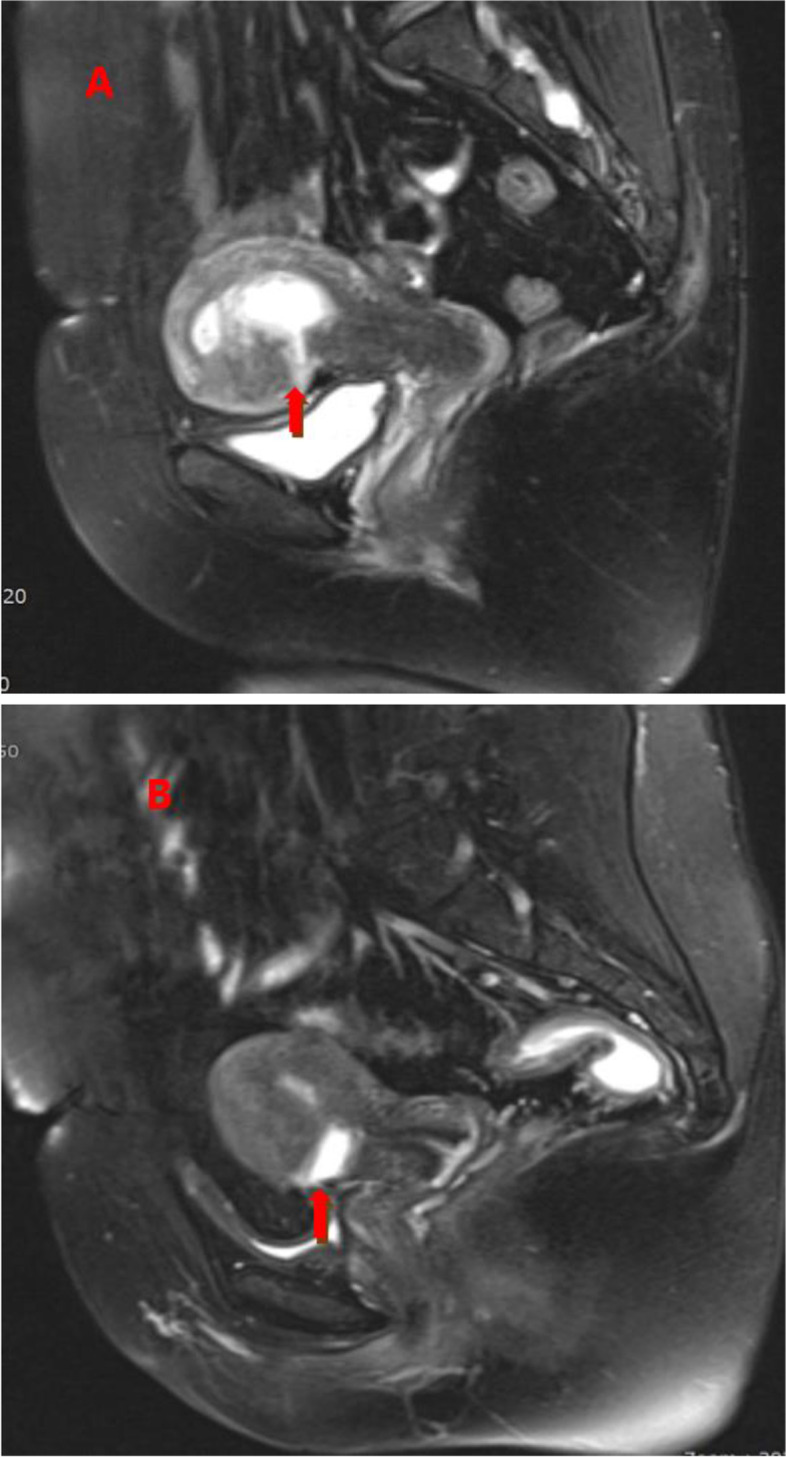
MR images of the patients with CSP. **A** MRI image shows the amniotic sac on the scar with progression of the pregnancy implanted in the isthmus uteri and in the uterine cavity (arrow). **B** MRI image shows the amniotic sac deeply implanted into previous cesarean scar defect with infiltrating growth into the uterine myometrium (arrow)

**Fig. 3:**
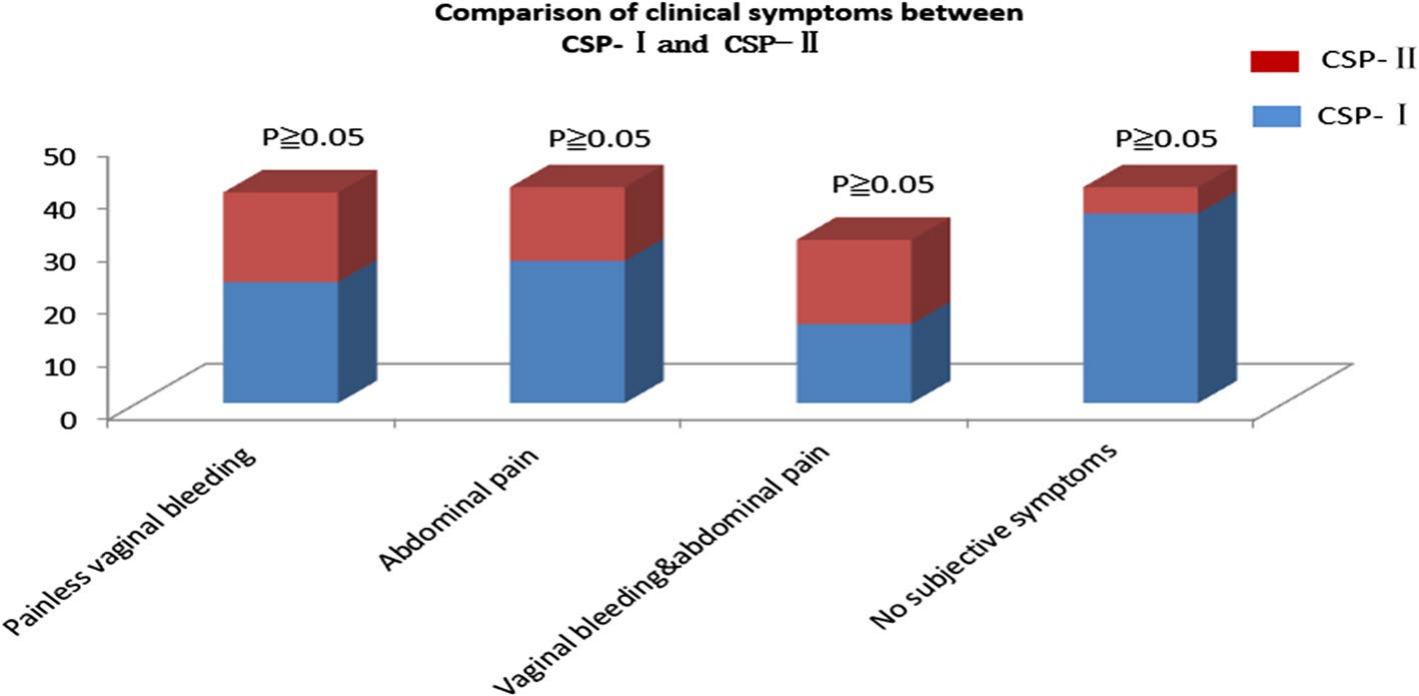
Comparison of clinical symptoms between CSP-I and CSP-II. The picture shows the distribution of main clinical symptoms and no subjective symptoms in CSP-I and CSP-II under the treatment of USg-D&C performed after HIFU ablation. The red means the distribution of CSP-I, the blue means the distributions of CSP-II

There were 153 patients with CSP identified for this study. Among the patients enrolled in this study, 101 had CSP-I, and 52 had CSP-II (Fig. 2). Data on the baseline characteristics of the two types were collected (Table 1). There were no significant differences in maternal age, gestational day, gestational sac/mass diameter, number of cases with detected fetal heart activity and the β-HCG level. However, the thickness of the myometrium between the gestational sac and the bladder was 3.93 ± 1.25 vs.2.55 ± 1.46 mm, respectively (*P <* 0.05). there were 23,27,15 and 36 painless vaginal bleeding, abdominal pain, vaginal bleeding lower abdominal pain and asymptomatic case respectively. In the CSP-IIgroup, there were 17, 14 16 and 5 painless vaginal bleeding, abdominal pain vaginal bleeding with lower abdominal pain and asymptomatic case, respectively. There was no statistical difference in the clinical symptoms of two groups (Fig. 3).

**Table 1:** The baseline characteristics of patients with CSP-I and CSP-II group

Characteristics	CSP-I(*n =* 101)	CSP-II(*n =* 52)	*p* value
Age (year)	31.56 ± 4.05	33.61 ± 4.46	0.231
Gestational days (day)	55.75 ± 6.13	57.85 ± 5.19	0.457
Pretreatment HCG (mIU/ml)^a^	37,438.10 (154.5-65,277)	32,688.63 (71-54,962)	0.692
Gestational sac/mass diameter (mm)	35.17 ± 7.05	36.12 ± 8.60	0.544
Myometrium thickness (mm)	3.93 ± 1.25	2.55 ± 1.46	0.032
Fetal heart activity detected(n)^b^			0.121
Yes	58	23	
NO	43	29	

Data are presented as mean ± SD, except for “Pretreatment HCG and fetal heart activity detected”

^a^Pretreatment b-HCG listed as medians

^b^The numbers for fetal heart activity detected are listed according to respective groups

### HIFU ablation followed by USg-D&C evaluation

The success rate was defined as the efficiency of first-line treatment; complications rate hysterectomy and/or hemorrhage≥1000 ml [12]. All patients completed the treatment successfully without hysterectomy and/or severe hemorrhage in our study. Detailed HIFU ablation results are shown in Table 2. The average total treatment time defined from the first energy exposure to last energy exposure. In the CSP-I group, the median total treatment time, median sonication time and median sonication power were 73 min, 583 seconds and 367 w, respectively. In the CSP-II group, the median total treatment time, median sonication time and median sonication power were 96 min, 601 seconds and 388 w, respectively. The length of median total treatment time of HIFU ablation for the CSP-II group was significantly longer than that for the CSP-I group (*P <* 0.05). Before HIFU ablation, ultrasound showed fetal cardiac activity in 81 cases of two groups. After HIFU ablation, fetal cardiac activity disappeared. Three to 6 days after HIFU ablation, 153 patients underwent USg-D&C. The average intraoperative blood loss of USg-D&C was 39 ml in the CSP-I group and 65 ml in the CSP-II group (*P <* 0.05), the average USg-D&C time was 29 vs.45 min for the CSP-I and CSP-II respectively (*P <* 0.05).

**Table 2:** The results of HIFU followed by USg-D&C comparison between CSP-I and CSP-II group

Characteristics	CSP-I(*n =* 101)	CSP-II(*n =* 52)	*p* value
HIFU treatment			
Total treatment time (min)	73 (52- 147)	96 (67-153)	0.038
Sonication time (second)	583 (426-716)	601 (469-798)	0.217
Sonication power(W)	367 (350-400)	388 (350-400)	0.862
USg-D&C			
USg-D&C time (min)	29 (20-49)	45 (25-70)	0.037
Median blood loss (ml)	39 (15-100)	65 (30-210)	0.021
Post-treatment of β-HCG (mIU/ml)	13,852.38 (90.5-24,727)	9368.6 (56-26,825)	0.571
Time of hospital stay (days)	7.07 ± 1.83	7.18 ± 1.72	0.620
Vaginal bleeding of post-treatment (day)	15.3 ± 3.6	16.1 ± 4.3	0.821
Time for β-HCG return to normalization (day)	26.08 ± 5.02	28.03 ± 4.99	0.652
Recovered of menstruation (day)	38.22 ± 6.15	36.16 ± 5.57	0.471

Total treatment time, sonication time, sonication power, USg- D&C time, median blood losses and post-treatment of β-HCG are listed as medians

### Adverse effects and complications

After HIFU treatment, 81 patients complained of lower abdominal or pelvic pain in the treated area, 68 patients complained of lower limb pain numbness, 26 patients complained of sacrum pain,31 patients complained of fever postoperative were noted. However, the difference between the two groups was not statistically significant (*P* > 0.05) (Table 3). No serious complications such as bladder injury, skin injury and intestinal injury occurred.

**Table 3:** The adverse effects and complications of HIFU ablation between the CSP-I and CSP-II group

Characteristics	CSP-I(*n =* 101)	CSP-II(*n =* 52)	*p* value
Lower abdominal or pelvic pain(n)	58	23	0.128
Lower limb pain numbness(n)	49	19	0.158
Sacrum pain(n)	18	8	0.702
Fever(n)	21	10	0.820

### Follow-up results

All patients successfully completed treatment. The follow-up period was 15.3 ± 5.6 months. The duration of hospitalization was 7.07 ± 1.83 days in the CSP-I group and 7.18 ± 1.72 days in the CSP-II group (*P* > 0.05). The postoperative β-HCG level was 13,852.38 (90.57-24,727) mIu/ml in the CSP-I group and 9368.6 (56-26,825) mIu/ml in the CSP-II group, and no significant difference was observed. The patients were recommended to have their blood β-HCG level test once a week until they returned to normal level. The average time needed for β-HCG return to normal levels was 26.08 ± 5.02 days in the CSP-Igroup and 28.15 ± 4.99 days in the CSP-IIgroup (*P* > 0.05). The duration of vaginal bleeding was 15.3 ± 3.6 days in the CSP -I group. and 16.1 ± 4.3 days in the CSP-II group after USg-D&C, (*P >* 0.05), and the menstruation of the CSP-I and CSP-II patients recovered 38.22 ± 6.15 and 36.16 ± 5.57 days after HIFU ablation, respectively (*P >* 0.05). No patient had amenorrhea. During the follow-up period, 9 patients conceived,4 patients were in the CSP-II group, 5 patients were in the CSP-1 group. 6 patients delivered babies at full-term underwent cesarean section, and 1 patient was still pregnancy, while 1 patient suffered from CSP again in CSP-II, 1 patient had elective terminations in the first trimester.

## Discussion

CSP is a long-term complication of cesarean section that carries a high-risk of uncontrolled hemorrhage. The incidence of CSP was reported from 1/1800 to 1/2216 in pregnancies, and account for 6.1% of all ectopic pregnancies in patients who had at least one cesarean section [13]. Over the last 10 years, the incidence of CSP has been increasing because of the increased cesarean delivery rate and the advances of diagnosis in ultrasound and MRI [3, 4]. However, there is still no consensus guideline for the management of CSP, more than 30 therapeutic schedules for patients with CSP, including systemic/ local administration Methotrexate (MTX), uterine artery embolization (UAE) followed by curettage, removal of the CSP transvaginal, laparoscopically or assisted by hysteroscopy have been reported [14, 15]. Each of therapy has its own individual advantage and disadvantage. Yang H et al. found that methotrexate administration could significantly improve the curative effect of cesarean section patients with scar pregnancy by taking 160 patients with scar pregnancy as research subjects [16]. However, methotrexate is not suitable for patients with high HCG levels or patients with fetal cardiac activity; Slow onset after initial administration; Causes complications such as bone marrow suppression and digestive system symptoms; The serum β-HCG level became negative for a long time. In another study, anhydrous ethanol chorionic villus targeting therapy was also an alternative option compared to methotrexate administration. Inject anhydrous ethanol into the sac directly could kill trophoblast cells, and level of β -HCG decreased significantly within a month [17]. However, when anhydrous ethanol leaks into the abdominal cavity, peritoneal irritation occurs, causing hematoma around the pregnancy tissue. It has been reported that CSP resection under hysteroscopy is a safe and effective minimally invasive treatment. The hysteroscopy passes through the vagina into the uterus, visually identifying and scraping out pregnancy tissue. This method can cause little damage to the endometrium and has little impact on fertility [18]. However, if the residual pregnancy tissue is still active, the persistent may lead to persistent ectopic pregnancy. Postoperative complications such as intrauterine adhesion, oligomenorrhea, even amenorrhea and menstrual bleeding can occur, which seriously affect the quality of life of patients [19]. In the study of Pyra K et al., UAE was shown to be a safe and effective method with the advantages of timely hemostasis, low trauma and high success rate, and should be considered as an option for CSP treatment, especially for women who wish to maintain fertility [20]. However, in another study, UAE caused platelet aggregation, fibrin deposition and thrombosis [21]. In PyraK's follow-up study, a patient suffered from menstrual insufficiency, which may be due to utero-ovarian artery anastomosis. After uterine artery embolization, ovarian blood supply was affected, resulting in ovarian necrosis. HIFU adopts a non-invasive method to ablate the pregnancy tissue, which causes the necrosis of the pregnancy tissue and is conducive to subsequent curettage, reducing the residual pregnancy tissue and reducing vaginal bleeding. After treatment, the patient's main complaint was lower abdomen or lumbosacral pain, which was relieved within 1 week without special treatment [22]. In summary, HIFU treatment of CSP is safer and more effective than other methods. The ideal treatment strategy of CSP should meet the following criteria: safety, effectiveness, and/or a quick recovery of menstruation and fertility [11]. To date, several researches reported and manifested that HIFU ablation was safe for patients with CSP, but there is no report the results about the different types patients of CSP [5–8]. This study showed that the average time for gestational sac disappeared, vaginal bleeding of post-treatment, β-HCG level reduction to normal level, normal menstruation recovery and hospital stay were was not significantly different between CSP-Iand CSP-II. The safety of this non-invasive technique in the treatment of CSP patients is always a concern. Complication of HIFU ablation including skin burns in the treatment, fever,abdominal or pelvic pain, and distension-radiating pain into the lower limbs, have been described in reports on the experiment of treatment of uterine fibroids [15, 23, 24]. In this study, immediately after HIFU ablation, the common adverse effect was lower abdominal or pelvic pain. There were no statistical differences in the adverse effects between the two groups. During the follow-up, nine patients became pregnant again. Fertility is affected by variety of factors including the maternal age, and ovarian reserve. Because of the small number of patients, the study did not analyze the potential factors in successful pregnancy after HIFU treatments in the two types CSP. The recovery of normal menstruation and conception during follow-up period of patients with CSP demonstrated that HIFU ablation combined with USg-D&C treatment for CSP has less adverse and is beneficial to retain the further fertility function. The great challenge in the treatment of HIFU ablation combined with USg-D&C for CSP is the anatomical position of the pregnancy lesion, where the myometrium of embryo implanted is thin or even defect and increasing risk of severe bleeding. There was a statistically significant difference in the thickness of myometrium in anterior lower uterine part between the CSP-Iand CSP-IIin the study. Compared with CSP-I, CSP-II has greater potential risks of severe bleeding. Zhu et al. have treated 53 patients with CSP with suction curettage under hysteroscopic guidance after HIFU ablation, and the median volume of blood loss in the procedure 20 ml [7]. In a comparison study, Hong retrospective analyzed 152 CSP patients, who were treatment with HIFU ablation or UAE followed by hysteroscopy. Their results showed that blood loss was 76.38 ± 22.89 ml in the HIFU group, whereas it was 114.42 ± 30.34 ml in the UAE group. Zhang et al. reported that 25 CSP-II patients who were treated with transvaginal surgical management. The average intraoperative blood loss was 60.5 ml [22]. The intraoperative blood loss of the CSP-Iwas significantly less than that of CSP-IIin this study, without hysterectomy and hemorrhage≥1000 ml. This result indicated that HIFU ablation followed by USg-D&C is safety in the CSP-Iand CSP-II, and it seems to be superior to UAE, and similar to transvaginal surgical management, but less invasive. Pregnancy in the scar from a cesarean delivery is located outside or inside of lower uterine cavity and is completely or partial surrounded by myometrium and fibrous tissue of the scar in the prior low uterine segment [14]. Therefore, the scar surface of the lower anterior uterine wall may be deficient because of poor vascularity, fibrosis, and impaired healing. The objection of the management for CSP is to expel the pregnancy tissue in cesarean scar, decrease the sever bleeding risk. However, due to villus implanting in the muscular layer of lower uterine and lacking of effective shrinkage, directly curettage is not a first-line therapeutic option for CSP, because it could cause blood vessels rupture and catastrophic hemorrhage of uterus [25]. How to effectively reduce the blood supply of pregnancy tissue before D&C is a current direction of CSP treatment. The application of HIFU ablation, a noninvasive technique, was approved by the U.S. Food and Drug Administration (FDA) and modified in2004 [9]. The targeted tissue ablaion was achieved by instantaneous temperature elevation to 60–100°C, utilizing the physical characteristics of tissue penetration under the low-energy ultrasound waves which was produced by the HIFU treatment system [8, 9]. According to literature reports, the advantages of HIFU ablation CSP may be as follows: 1) a rapid decline of β-HCG level and cessation of embryonic cardiac activity; 2) a reduction of blood flow in the trophoblast tissue ultrasound assessment; 3) an apparently decreased the risk of hemorrhage during the D&C procedure [5–8, 22]. This study is limited because there is no international classification standard for CSP, and the special types of CSP have not been discussed in this research. It analyzed the safety and feasibility of HIFU ablation followed by USg-D&C for two types of CSP, but did not compare to other method, such as MTX or UAE. This study suggested that HIFU, a non-invasion treatment, can appear to be superior as it decreased the risk of hemorrhage during the D&C procedure for two types CSP, which is a single-center retrospective study and the multicenter and prospective studies be necessary to validate our findings in the future.

## Conclusions

HIFU ablation followed by USg-D&C was safe and effective in treating the CSP-I and CSP-II patients, which may be a potential better noninvasive therapy option for patients with CSP. At present, the efficacy of HIFU ablation followed by USg- D&C treatment on the quality of life of the patients with CSP has few prospective studies. Besides, the therapeutic effect evaluation was obtained by the means of follow-up, which is  subjective. However, as a non-invasive treatment, HIFU was used as an adjunctive treatment in this study. Perhaps, with the improvement of technology in the future, HIFU will be used as a separate method for caesarean scar pregnancy treatment. 

## Data Availability

The datasets generated and/or analyzed during the current study are not publicly available, but are available from the corresponding author on reasonable request. Focused ultrasound ablation technology is a clinical project jointly carried out by our hospital and Chongqing Medical University, and related research is still under study. In this study, the pregnancy data of cesarean section scar incision treated by focused ultrasound ablation were selected to write the paper. According to the agreement of the research association and the requirements of intellectual property rights, the clinical data are not open to the public at present.

## Abbreviations

HIFU: High Intensity Focused Ultrasound
USg-D&C: Ultrasound-guided Dilation and Curettage (USg-D&C)
CSP: Cesarean Scar Pregnancy (CSP)
CSP-I: Cesarean Scar Pregnancy I
CSP-II: Cesarean Scar Pregnancy II
SCH: Suining Central Hospital (SCH)
MTX: Methotrexate
UAE: Uterine Artery Embolization
FDA: the U.S. Food and Drug Administration
